# Synthesis of Thiazolidin-4-Ones Derivatives, Evaluation of Conformation in Solution, Theoretical Isomerization Reaction Paths and Discovery of Potential Biological Targets

**DOI:** 10.3390/molecules29112458

**Published:** 2024-05-23

**Authors:** Nikitas Georgiou, Danai Karta, Antigoni Cheilari, Franci Merzel, Demeter Tzeli, Stamatia Vassiliou, Thomas Mavromoustakos

**Affiliations:** 1 Laboratory of Organic Chemistry, Department of Chemistry, National and Kapodistrian University of Athens, Panepistimioupolis Zografou, 11571 Athens, Greece; nikitage@chem.uoa.gr (N.G.); danaikarta@gmail.com (D.K.); svassiliou@chem.uoa.gr (S.V.); 2 Department of Pharmacognosy and Natural Products Chemistry, Faculty of Pharmacy, National and Kapodistrian University of Athens, Panepistimiopolis Zografou, 15771 Athens, Greece; cheilarianti@pharm.uoa.gr; 3 Theory Department, National Institute of Chemistry, 1000 Ljubljana, Slovenia; franci.merzel@ki.si; 4 Laboratory of Physical Chemistry, Department of Chemistry, National and Kapodistrian University of Athens, Panepistimioupolis Zografou, 11571 Athens, Greece; tzeli@chem.uoa.gr; 5 Theoretical and Physical Chemistry Institute, National Hellenic Research Foundation, 48 Vassileos Constantinou Ave., 11635 Athens, Greece

**Keywords:** thazoline, NMR, DFT, molecular docking, molecular dynamics, drug-likeness

## Abstract

Thiazolin-4-ones and their derivatives represent important heterocyclic scaffolds with various applications in medicinal chemistry. For that reason, the synthesis of two 5-substituted thiazolidin-4-one derivatives was performed. Their structure assignment was conducted by NMR experiments (2D-COSY, 2D-NOESY, 2D-HSQC and 2D-HMBC) and conformational analysis was conducted through Density Functional Theory calculations and 2D-NOESY. Conformational analysis showed that these two molecules adopt *exo* conformation. Their global minimum structures have two double bonds (C=N, C=C) in Z conformation and the third double (C=N) in E. Our DFT results are in agreement with the 2D-NMR measurements. Furthermore, the reaction isomerization paths were studied via DFT to check the stability of the conformers. Finally, some potential targets were found through the SwissADME platform and docking experiments were performed. Both compounds bind strongly to five macromolecules (triazoloquinazolines, mglur3, Jak3, Danio rerio HDAC6 CD2, acetylcholinesterase) and via SwissADME it was found that these two molecules obey Lipinski’s Rule of Five.

## 1. Introduction

Thiazolidin-4-ones, reduced forms of thiazole, represent an important heterocyclic ring system and are important scaffolds in medicinal chemistry. They display numerous activities, such as antimicrobial [1], anticancer [2], antioxidant [3], anti-inflammatory and analgesic [4], antidiabetic [5], antiparasitic [6] and antiviral [7].

The thiazolidin-4-one ring is susceptible to modifications in positions 2, 3 and 5. Such modifications capacitate the search for new compounds with desired activities. In particular, 5-substituted thiazolidin-4-ones derive from synthetic procedures applied to the methylene carbon. Knoevenagel reaction is one of them and has been applied to introduce a substituent in 5-position [8], introducing a double bond, usually of *Z*-configuration [9].

In our continuous effort towards the synthesis of new pharmacologically interesting scaffolds, we synthesized two 5-substituted thiazolidin-4-ones (**DKI39, DKI40**). The stereochemical outcome of these compounds, possessing three double bonds, was studied extensively with 2D NMR experiments and computational chemistry, leading to their unambiguous conformational characterization. Specifically, Density Functional Theory (DFT) was used to calculate the lowest energy conformer. The lowest energy conformer was superimposed with the experimental conformation derived using 2D NOESY spectroscopy. If the RMSD of the superimposition was found to be less than 2Å, then, experimentally, the two conformations were considered to be identical. Furthermore, the reaction isomerization paths were studied via DFT to check their stability.

Finally, as a continuation of our previous studies, where similar molecules presented strong binding affinity in macromolecules [10,11], the present compounds were tested through in silico molecular docking in different macromolecules to check if the present compounds are suitable for specific biological targets. The results gave promising bindings and SwissADME showed that both compounds obey Lipinski’s Rule of Five. To the best of our knowledge, this is the first study towards this end.

## 2. Results

### 2.1. Synthesis

**Scheme 1:**
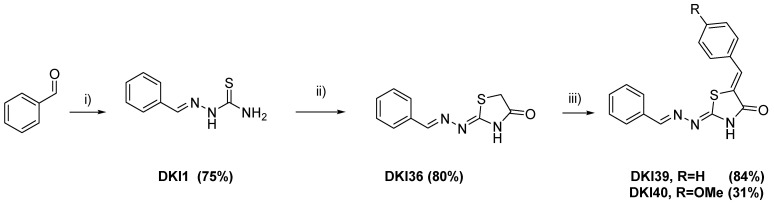
Reagents and conditions: (**i**) thiosemicarbazide, EtOH, cat. AcOH, 80 °C, 4 h; (**ii**) methyl 2-chloroacetate, MeOH, CH_3_COONa, 65 °C, 6 h; (**iii**) (a) benzaldehyde, MeOH, cat. Piperidine, 65 °C, 48 h (for **DKI39**) and (b) anisaldehyde cat. piperidine, rt, 7d (for **DKI40**).

Thiosemicarbazone **DKI1** was synthesized from benzaldehyde and thiosemicarbazide under standard conditions and it was further transformed to thiazolidin-4-one **DKI36** using methyl 2-chloroacetate, following our previously published conditions.^11^ Knoevenagel reaction between **DKI36** and two aromatic aldehydes in the presence of piperidine provided 5-substituted thiazolidin-4-ones **DK139** and **DKI40**. For **DKI39**, a similar synthetic strategy has been reported in the literature [12] using **DKI36**, benzaldehyde and NaOAc in refluxing glacial acetic acid at the final step, but in our hands, this reaction failed to give the desired product. Moreover, Kambe [13] reported a totally different **DKI39** synthetic pathway using ethyl thiocyanoacetate as the starting material, providing the desired compound, albeit in very low yield (32%) (Scheme 1).

### 2.2. Structure Assignment

As a convenient starting point for structure elucidation of **DKI39**, the proton that resonates at 8.54 ppm was chosen. This peak corresponds to H-5, as it appears as a single peak, since it is not coupled with any other peak. Another important proton, the H-3 was identified as it gives a spatial correlation signal with H-5, through 2D-NOESY. Then, C-5 and C-3 were also identified, as they give ^1^J_C-H_ correlation with H-5 and H-3, respectively, through 2D-HSQC. Through 2D-COSY, H-2 and H-1 were identified as they give a correlation signal with H-3. Specifically, H-2 was identified because it gives triplet peak. C-2 and C-1 were then also identified, as they give ^1^J_C-H_ correlation with H-2 and H-1. H-12 was immediately identified, as it is the only proton that gives a correlation signal with C-10 of the carbonyl, through 2D-HMBC. After the identification of H-12, H-14 was identified as it gives a spatial correlation with H-12, through 2D-NOESY. H-15 and H-16 were also immediately identified, as they give a correlation signal through 2D-COSY. H-15 was identified, because it gives a pair of doublet peaks as it correlates with two protons. C-15, C-16, C-12 and C-14 were also identified, since they give a correlation signal with these protons. Through 2D-HSQC of the examined molecules, all carbons were identified except quaternary and carbonyl carbons. The remaining carbons could be identified through 2D-HMBC. Specifically, it was observed that H-5 showed ^2^J_C-H_ correlation with C-4, H-12 showed ^2^J_C-H_ and ^3^J_C-H_ correlation with C-10 and C-11, respectively, and H-15 showed ^3^J_C-H_ with C-13.

**Scheme 2:**
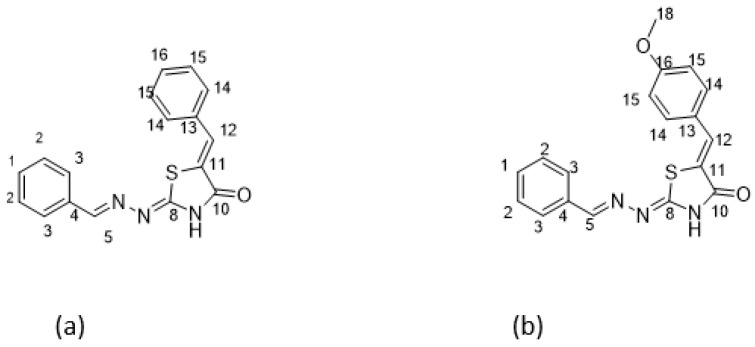
Structures of exo conformers of **DKI39** (**a**) and **DKI40** (**b**).

A similar procedure was carried out for thiazolidinone **DKI40**. The two structural identification strategies are shown in Supporting Information. The structures of **DKI39** and **DKI40** are shown in Scheme 2 with their numbering. The chemical shifts for both compounds are shown in Table 1.

### 2.3. Conformational DFT Analysis

**Scheme 3:**
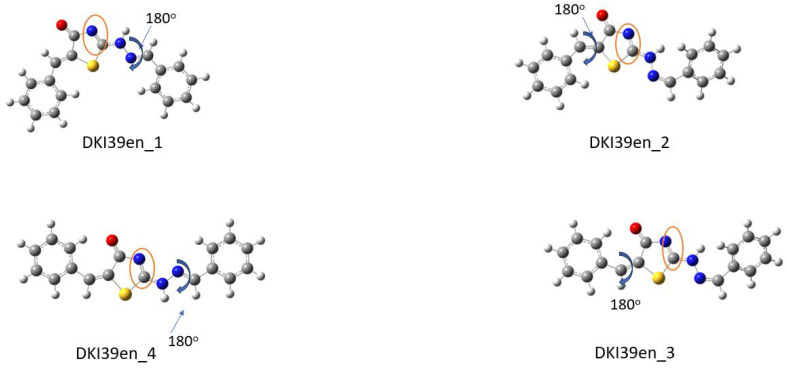
Optimized conformations derived from DFT calculations for the **DKI39** *endo* compound. In the Figure the dihedral angles rotated by 180° are shown. For instance, in DKI39en_1, the dihedral angle τ_1_ was rotated and DKI39en_2 was obtained. In the orange circle, the double bond that indicates the *endo* nature of compound is shown.

DFT was used to predict the lowest minima for both compounds. Various initial structures were geometry-optimized in order to calculate the lowest energy isomers (*E/Z*) and their conformers (*endo/exo*). Exo conformers are shown in Scheme 2, while endo conformers are shown in Scheme 3. In the endo form, the double bond is between C8 and N next to the carbonyl, while in the exo form, the double bond is between C8 and N7. Three dihedral angles were selected for each compound. Specifically, for **DKI39** the angles that were selected are formed by the following atoms: 4-5-6-7 (τ_1_), 10-11-12-13 (τ_2_) and 6-7-8-9 (τ_3_), and for **DKI40**: 4-5-6-7 (τ_1′_), 6-7-8-9 (τ_2′_) and 10-11-12-13 (τ_3′_). The conformations DKI39en_1 and DKI40en_1 were used as initial structures. Then, by rotating one dihedral angle each time by approximately 180°, the next conformation was occurred. For instance, starting from DKI39en_1, if τ_1_ is rotated by 180°, DKI39en_2 will be obtained (Scheme 3). Also, starting from DKI39en_2, if τ_1_ and τ_2_ is rotated by 180°, DKI30en_3 will be obtained, as it is shown in Scheme 3. All conformers were geometry-optimized.

**Scheme 4:**
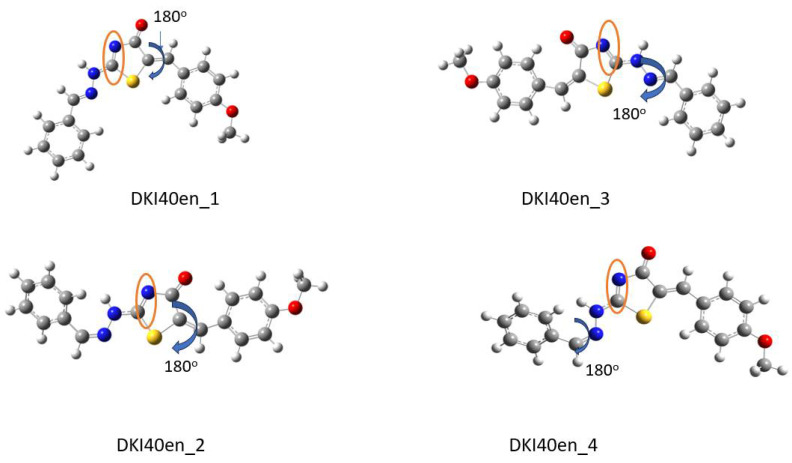
Optimized conformations derived from DFT calculations for the **DKI40** *endo* compound. In the Figure, the dihedral angles rotated by 180° are shown. For instance, in DKI40en_1, the dihedral angle τ_3′_ was rotated and DKI39en_2 was obtained. In the orange circle, the double bond that indicates the *endo* nature of compound is shown.

Considering the *endo* compound for **DKI39** and **DKI40**, the conformations that occurred are showed in Scheme 3 and Scheme 4. The double bond, which is considered as *endo* for both of the compounds, is shown in orange circle, and it is the same for the four different conformations.

**Scheme 5:**
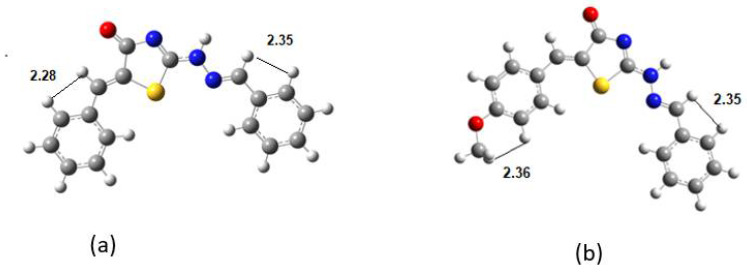
The global minima conformations for (**a**) **DKI39** and (**b**) **DKI40** *endo* compounds.

The relative energies are given in Table 2 for the *endo* isomorph with the values of dihedral angles for both compounds. Considering the energetics values and the critical distances obtained from NMR experiments, the DKI39en_1 and DKI40en_1 structures are the most favorable isomers for the *endo* compounds. The structures are shown below in Scheme 5.

**Scheme 6:**
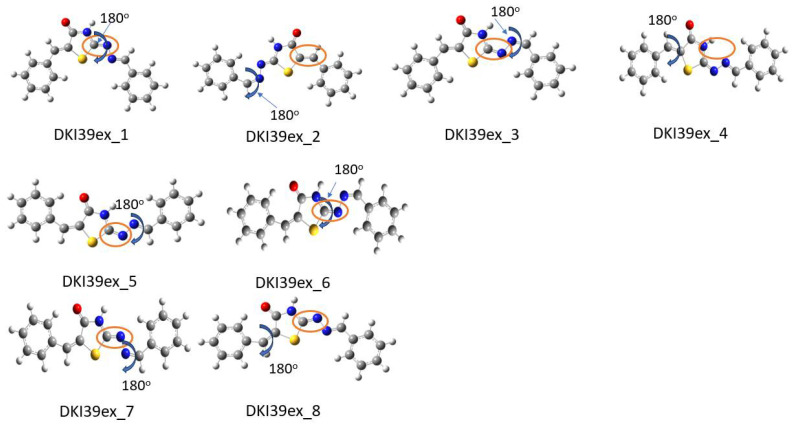
Optimized conformations derived from DFT calculations for **DKI39** *exo* compound. In the Figure the dihedral angles rotated by 180° are shown. For instance, in DKI39ex_1, the dihedral angle τ_3_ was rotated and DKI39ex_2 was obtained. In the orange circle, the double bond that indicates the *exo* nature of compound is shown.

**Scheme 7:**
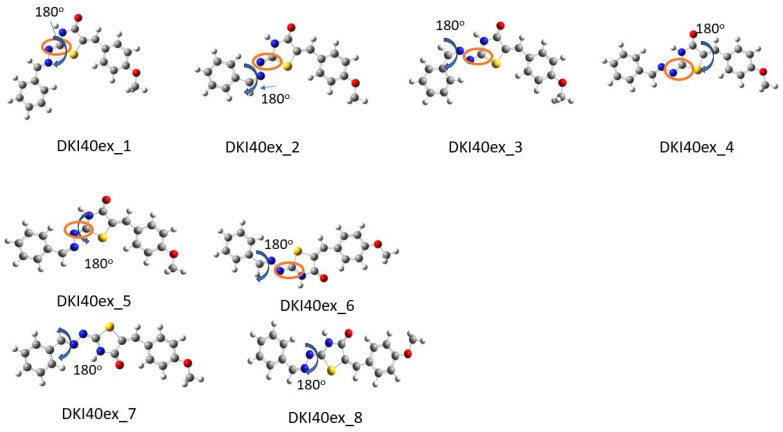
Optimized conformations derived from DFT calculations for the **DKI40** *exo* compound. In the Figure, the dihedral angles rotated by 180° are shown. For instance, in DKI40ex_1, the dihedral angle τ_2′_ was rotated and DKI40ex_2 was obtained. In the orange circle the double bond that indicates the *exo* nature of compound is shown.

The conformations DKI39ex_1 and DKI40ex_1 were the initial optimized structures. Then, by rotating the dihedral angles by 180°, additional conformations were obtained, which subsequently were geometry-optimized. For example, from DKI39ex_1 to DKI39ex_2, τ_1_ dihedral angle was rotated by 180° approximately. Considering the *exo* compounds, the conformations that occurred for **DKI39** and **DKI40** are shown in Scheme 6 and Scheme 7. The double bond, which is considered as *exo* for both compounds, is shown in a orange circle and it is the same for the eight different conformations. The relative energies are given in Table 3 for the *exo* isomorph, as well as the values of dihedral angles for both compounds. In the next section, the transition states are presented.

**Scheme 8:**
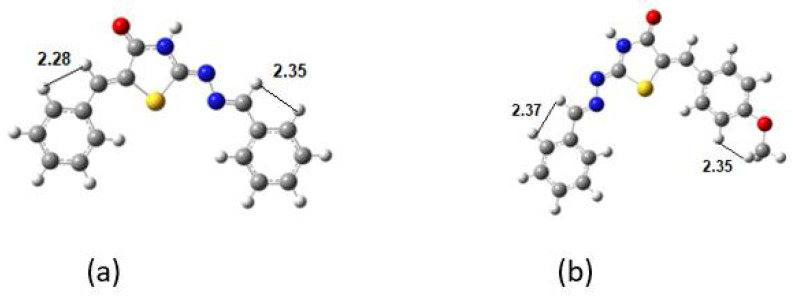
The lowest in energy conformations for (**a**) **DKI39** and (**b**) **DKI40** *exo* compounds.

**Figure 1:**
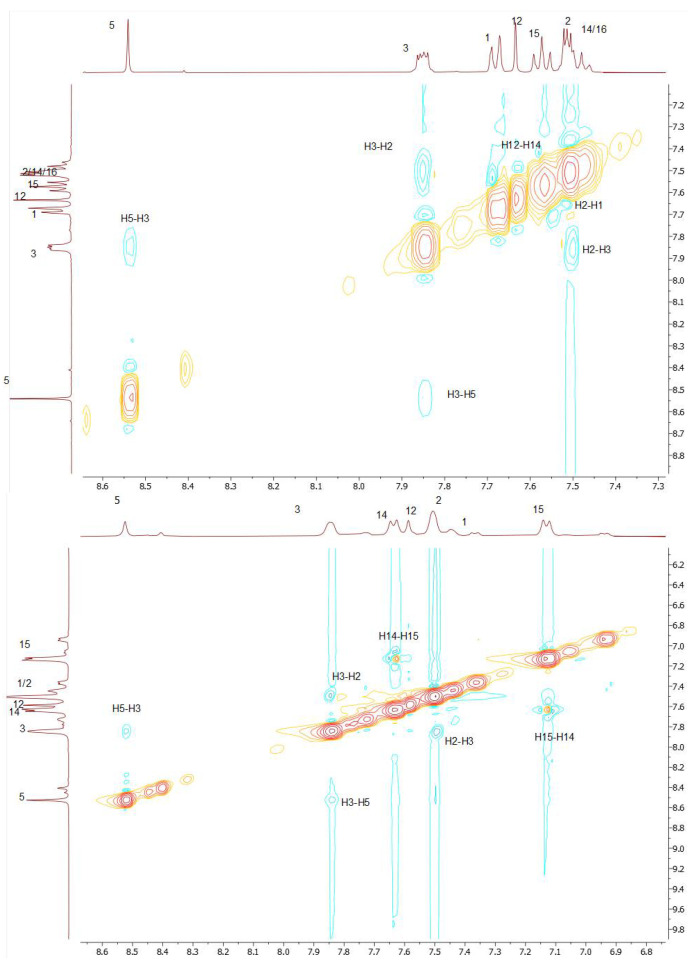
NOE effects for **DKI39** (**top**) and for **DKI40** (**bottom**).

The dihedral angles of the minima were expected to be about 180° and 0°, however, there are deviations from these values due to stereochemical hindrances within the structures. The lowest energy conformations for **DKI39** and **DKI40** *exo* compounds are DKI39ex_1 and DKI40ex_1, respectively. To sum up, the DFT calculations show that the *exo* isomer is lower in energy than the *endo* one for both molecules. Specifically, among the *exo* calculated conformations, the DKI39ex_1 and DKI40ex_1 are the energetically more favorable isomers of the **DKI39** and **DKI40** compounds (Scheme 8). In particular, the *endo* conformer is higher than *exo* by 3 kcal/mol approximately. This was also confirmed because no spatial relationship was observed in 2D-NOESY between the NH group with the H-5 in both molecules (Figure 1). Also, in the *exo* conformers, there is an extended resonance which cannot be applied on the higher energy *endo* conformer. In Table 4, selected distances of the most energetical favorable conformations which have also been observed in 2D-NOESY are shown (Figure 1).

### 2.4. Isomerization Reaction Paths

**Figure 2:**
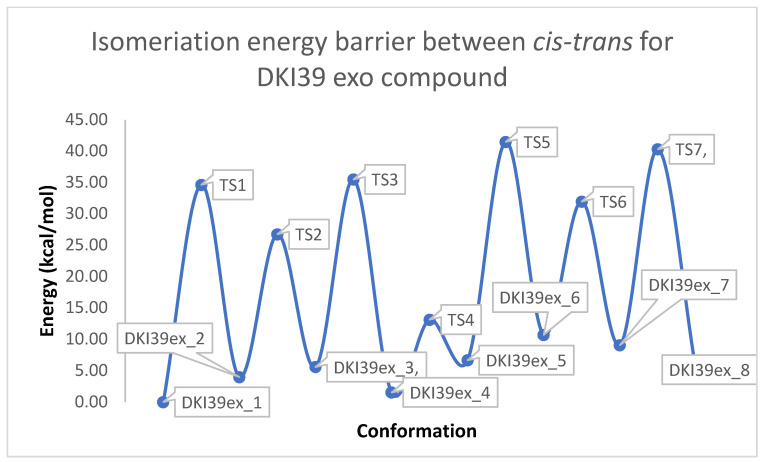
Schematic representation of the isomerization energy barrier between *cis-trans* for **DKI39** (y = energy in kcal/mol, x = conformation).

**Figure 3:**
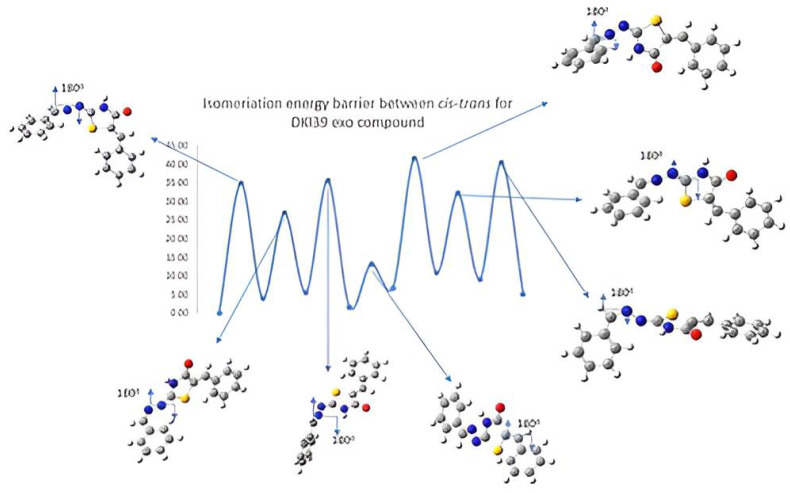
Schematic representation of *cis-trans* kinetic isomerization for **DKI39** *exo* compound (y = energy in kcal/mol, x = conformation). In each transition state, the dihedral angle, which is rotated by ~180°, is shown by an arrow.

**Figure 4:**
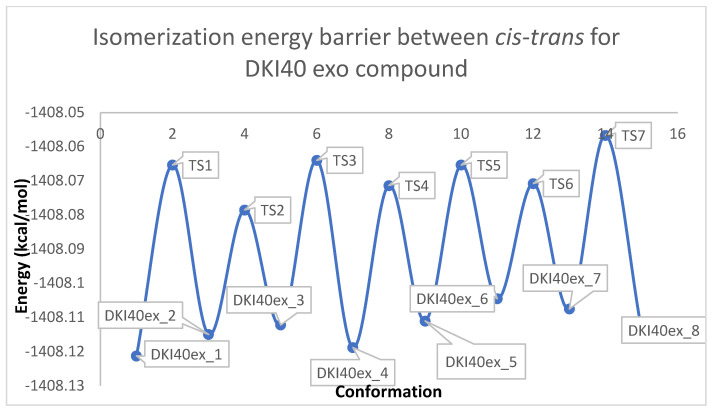
Schematic representation of the isomerization energy barrier between *cis* −*trans* for **DKI40** (y = energy in kcal/mol, x = conformation).

**Figure 5:**
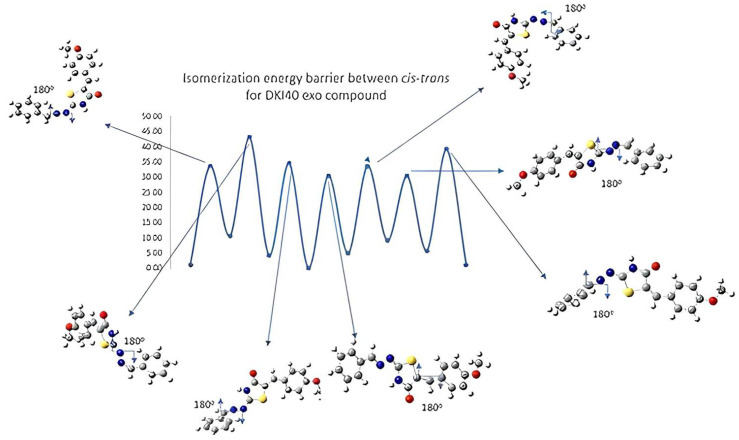
Schematic representation of *cis-trans* kinetic isomerization for **DKI40** *exo* compound (y = Energy in kcal/mol, x = conformation). In each transition state, the dihedral angle, which is rotated by ~180°, is shown by arrow.

The isomerization reaction paths, including the corresponding transition states that are involved in the reaction paths, were calculated [14]. The *cis-trans* isomerization of **DKI39** is plotted in Figure 2 and Figure 3 and *cis-trans* isomerization of **DKI40** is plotted in Figure 4 and Figure 5.

The DFT calculations show that the energy gaps are quite high, i.e., larger than 20 kcal/mol, showing that there is only one dominant conformation for both compounds and it is difficult for the isomerization process from one isomer to other to occur. This conclusion is in agreement with our results obtained from our 2D-NMR spectra.

### 2.5. Molecular Binding

Molecular docking calculations were performed for both compounds in order to find some possible biological targets. From SwissADME [15], five possible targets were found. The grid parameters used were the same for all the substrates—X = 40, Y = 40, Z = 40 (default)—and the distance of the dots was 0.375 Å (default).

The coordinates for the active site for each macromolecule were: 2Y0J [16]: X = −21.975, Y = −16.275, Z = 32.38, 5CNK [17]: X = 12.642, Y = −14.827, Z = 9.451, 5TTV [18]: X = 3.17, Y = 14.033, Z = −3.123, 8A8Z [19]: X = −11.45, Y = −9.124, Z = −4.464, 4EY7 [20]: X = −18.53, Y = −41.928, Z = 24.258. The results from docking experiments are shown in Table 5.

Regarding the results, it was observed that in all macromolecules, the compounds bind strongly to the active site. Only in macromolecule mglur3 compound did **DKI40** not bind strongly to the active site.

**Figure 6:**
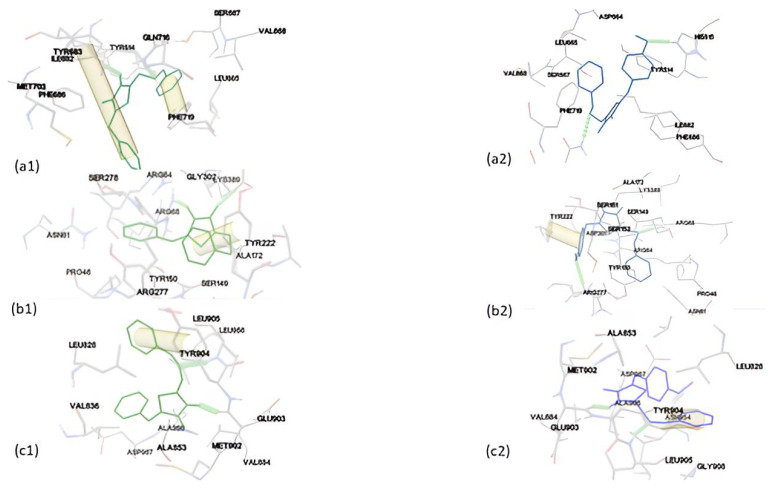

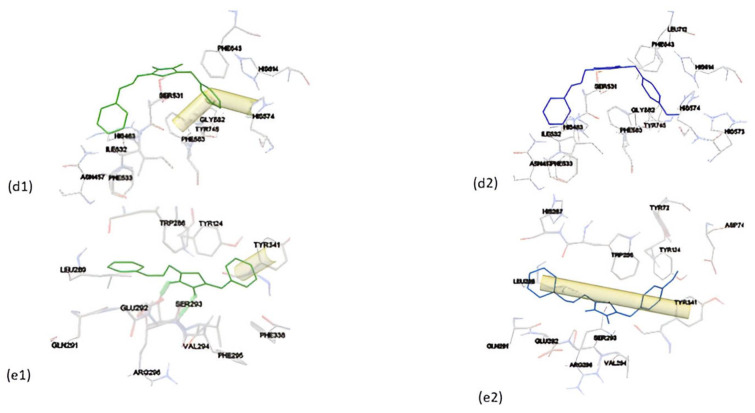
Binding mode of **DKI39** (left, green color) with (**a**) triazoloquinazolines, (**b**) mglur3, (**c**) Jak3, (**d**) Danio rerio HDAC6 CD2 and (**e**) acetylcholinesterase. Binding modes of **DKI40** (right, blue color) with (**a**) triazoloquinazolines, (**b**) mglur3, (**c**) Jak3, (**d**) Danio rerio HDAC6 CD2 and (**e**) acetylcholinesterase.

In Figure 6, all binding modes are shown for both compounds. Firstly, **DKI39** forms two hydrogen bonds with ILE682 and GLN716 and two π-π interactions with PHE719 and TYR683 of triazoloquinazolines. Also, **DKI39** forms two hydrogen bonds with ARG68 and ARG277 and one π-π interaction with TYR222 of mglur3. Moreover, **DKI39** forms one π-π interaction with TYR904 and two hydrogen bonds with LEU905 and GLU903 of Jak3. Furthermore, it forms two π-π interactions with HIS574 and PHE583 of Danio rerio HDAC6 CD2. Also, it forms one π-π interaction with TYR341 and two hydrogen bonds with VAL294 and GLU292 of acetylcholinesterase.

On the other hand, **DKI40** forms two hydrogen bonds with GLN716 and HIS515 of triazoloquinazolines. Secondly, it forms two hydrogen bonds with ARG68 and ARG277 and one π-π interaction with TYR222 of mglur3. Furthermore, **DKI40** forms two hydrogen bonds with VAL884 and LEU905 and one π-π interaction with TYR904 of Jak3. With the enzyme Danio rerio HDAC6 CD2, it does not form any interaction, while it forms one π-π interaction with TYR341 of acetylcholinesterase.

### 2.6. Molecular Dynamics

**Figure 7:**
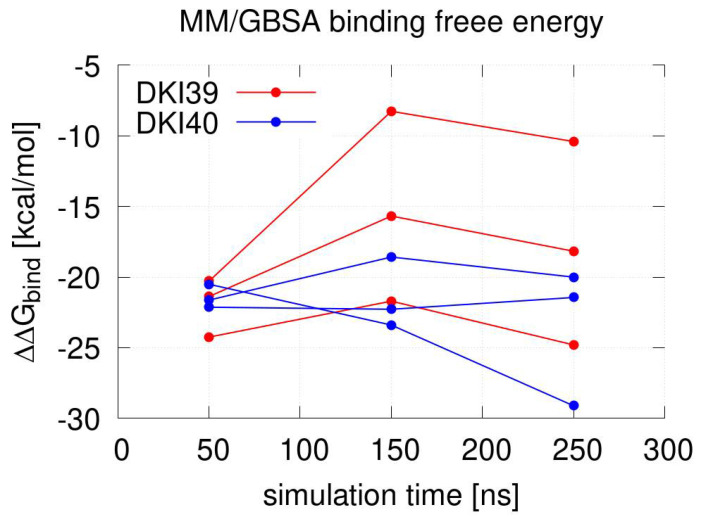
Comparison of binding free energies during repeated simulations (3 replicas) of **DKI39** and **DKI40** complexes in aqueous solution.

Molecular dynamics (MD) simulations were used to further elucidate binding of **DKI39** and **DKI40**. Three replica simulations, each of duration of 300 ns, were performed for each system, starting from the optimized conformations derived from DFT calculations. Due to its wide applicability and efficiency, we used the end-point calculation with molecular mechanics/generalized born surface area (MM/GBSA) [21] method of the binding free energy difference between the ligand, the corresponding protein receptor and the protein–ligand complex for both systems, ΔΔG_bind_ = ΔG_complex_ − ΔG_protein_ − ΔG_ligand_, where ΔG_complex_, ΔG_protein_ and ΔG_ligand_ are solvation free energies of the complex, protein and ligand, respectively. ΔΔGbind values were derived for the sequential intervals of 100 ns for each simulation and are shown in Figure 7. According to the negative values of ΔΔG, the simulation results indicate stable binding in both systems during MD simulations with a bit stronger interaction for **DKI40**.

### 2.7. Pharmacokinetics and Toxicity of the Compounds

The results from the drug-likeness of the compounds are shown in Table 6. According to SwissADME and pkCSM, both compounds obey Lipinski’s Rule of Five. [22] Due to the fact that rotable bonds are less than seven, the criterion for Veber’s Rule [23] is also met. Both compounds can be easily absorbed from the body because lipophilicity is less than 5.

Because the BBB [24] value (Table 7) is less than one, both compounds are inactive in the central nervous system (CNS). The value for human intestinal absorption is high for both compounds, and this signifies that these compounds might be better absorbed from the intestinal tract on oral administration. Both compounds are not inhibitors of CP isoenzymes and, therefore, are not toxic or do not exert other unwanted adverse effects.

According to preADMET, these are not hepatotoxic [25] and they have no skin sensitivity. But, the AMES toxicity is positive for both of them and that means that they may be mutagenic, see Table 8.

## 3. Materials and Methods

### 3.1. Experimental

Reagents were purchased with the highest commercial quality from Aldrich (St. Louis, MI, USA), Acros (Geel, Belgium) and Fluka (Buchs, Switzerland) and were used without further purification. Reactions were monitored by thin-layer chromatography (TLC) carried out on 0.25 mm silica gel plates (E. Merck silica gel 60F254), and components were visualized by UV light absorbance. Purification of compounds was performed with filtration. Mobile phase for TLC experiments was conducted with an appropriate mixture of PE [refers to petroleum ether (40–60 °C)] and EA [ethyl acetate], as described for each compound. Electrospray ionization (ESI) mass spectral analyses were performed on a mass spectrometer MSQ Surveyor, Finnigan, using direct sample injection. Negative or positive ion ESI spectra were acquired by adjusting the needle and cone voltages accordingly. HRMS spectra were registered using a 4800 MALDI-TOF mass spectrometer (Applied Biosystems, Foster City, CA, USA) in the positive reflection mode in the m/z range of 100–700.

#### 3.1.1. E-2-benzylidenehydrazinecarbothioamide, DKI1

To a mixture of benzaldehyde (4.0 mL, 40 mmol) in 95% ethanol (140 mL), thiosemicarbazide (3.65 g, 40 mmol) was added, followed by the addition of 4 drops of acetic acid. The reaction mixture was heated to 80 °C for 4 h. Then, to the reaction mixture, H_2_O (140 mL) was added and left to stand at 0 °C, the solid formed was filtered, washed with ethanol/water: 1/1 and dried to give **DKI1** as white solid in 75% yield. R_f_ (PE/EA: 2/1) = 0.69. Spectroscopic data are in accordance with the literature [26]. ^1^H NMR (600 MHz, DMSO-d_6_) δ: 7.38–7.43 (m, 3H), 7.79–7.81 (m, 2H), 8.00 (s, 1H), 8.06 (s, 1H), 8.21 (s, 1H), 11.44 (s, 1H) ^13^C NMR (101 MHz, DMSO-d6) δ: 127.82, 129.18, 134.70, 134.70, 142.79, 178.51 MS (ESI) calculated for C_8_H_10_N_3_S (Μ + H)^+^ 180.0 found 180.1.

#### 3.1.2. (Z)-2-(((E)-benzylidene)hydrazono)thiazolidin-4-one, DKI36

To a stirred solution of sodium acetate (6.89 g, 84 mmol) in MeOH (200 mL), thiosemicarbazone **DKI1** (7.6 g, 42 mmol) was added, followed by methyl 2-chloroacetate (4 mL, 42 mmol). The reaction mixture was warmed to 65 °C for 6 h in total, adding a new portion of methyl 2-chloroacetate every 2 h. The mixture was left to stand at room temperature, and the formed solid was filtered and washed with EtOH 95% to give **DKI36** as white solid in 80% yield. R_f_ = 0.54 (PE/EA 2:1) Spectroscopic data are in accordance with the literature [27]. ^1^H NMR (400 MHz, DMSO): δ 11.97 (s, 1H), 8.41 (s, 1H), 7.77–7.75 (m, 2H), 7.47–7.45 (m, 3H), 3.90 (s, 2H) ^13^C NMR (101 MHz, DMSO): δ 174.17, 165.35, 156.22, 134.20, 130.62, 128.82, 127.63, 33.02 MS (ESI) calculated for C_10_H_10_N_3_OS^+^ (Μ − H)^+^ 220.05 found 220.19.

#### 3.1.3. (Z)-2-(((E)-benzylidene)hydrazono)thiazolidin-4-one, DKI39

To a mixture of thiazolidine-4-one **DKI36** (219.3 mg, 1 mmol) in MeOH (2.5 mL), benzaldehyde (104 μL, 1 mmol) was added followed by piperidine (2 drops). The reaction mixture was warmed to 65 °C for 24 h, after which a new portion of benzaldehyde and piperidine was added, followed by 24 additional hours at 65 °C. The mixture was left to stand at room temperature, and the solid formed was filtered and washed with EtOH 95% to give **DKI39** as a pale yellow solid in 84% yield. R_f_ = 0.43 (PE/EA: 8:2).

^1^H NMR (400 MHz, DMSO): δ 12.63, (s, 1H/NH(9)), 8.53 (s, 1H/H-5), 7.85–7.83 (m, 2H/H-3), 7.68 (d, 1H, J = 7.5 Hz/H-1), 7.63 (s, 1H/H-12), 7.57 (t, 2H, J = 7.6 Hz/H-15), 7.52–7.49 (m, 3H/H-14,H-16), 7.43–7.45 (m, 2H/H-2) 13C NMR (101 MHz, DMSO): δ 178.34 (C-10), 167.33 (C-8), 157.85 (C-14), 133.84 (C-13), 133.56 (C-4), 131.05 (C-1), 129.88 (C-15), 129.83 (C-11), 129.27 (C-3), 129.14 (C-12), 128.89 (C-16), 127.97 (C-2), 122.99 (C-5). HRMS calculated for C17H14N3OS+ [M + H] 308.0852, found 308.0853.

#### 3.1.4. (Z)-2-(((E)-benzylidene)hydrazono)-5-((*Z*)-4-methoxybenzylidene)thiazolidin-4-one, DKI40

In a test tube, thiazolidinone **DKI36** (219.3, 1 mmol), anisaldehyde (496 μL, 4 mmol) and piperidine (2 drops) were mixed and stirred for 24 h at room temperature. In 24 h, a new portion of anisaldehyde and piperidine were added and the mixture was stirred for 7 days. The new solid formed was filtered and washed with EtOH 95% to give **DKI40** as a pale yellow solid in 31% yield. R_f_ = 0.58 (PE/EA 2:1).

^1^H NMR (400 MHz, DMSO): δ 12.63, (s, 1H/NH(9)), 8.53 (s, 1H/H-5), 7.85–7.83 (m, 2H/H-3), 7.68 (d, 1H, J = 7.5 Hz/H-1), 7.63 (s, 1H/H-12), 7,57 (t, 2H, J = 7.6 Hz/H-15), 7.52–7.49 (m, 3H/H-14,H-16), 7.43–7.45 (m, 2H/H-2), 3.86 (s, 3H/H-18)). 13C NMR (101 MHz, DMSO): δ 170.88 (C-10), 160.51 (C-8), 158.55 (C-14), 157.51 (C-16), 133.94 (C-13), 131.86 (C-4), 131.00 (C-1/C-5), 129.18 (C-15), 128.90 (C-11), 127.93 (C-3), 126.10 (C-12), 119.92 (C-16), 114.87 (C-2), 55.44 (C-18). HRMS calculated for C18H16N3O2S+ [M + H] 338.0958, found 338.0957.

### 3.2. Structure NMR Elucidation

The identification for both molecules was carried out via NMR using a 400 MHz spectrometer. Specifically, 1D (^1^H, ^13^C) and 2D (2D-COSY, 2D-NOESY, 2D-HSQC, 2D-HMBC) NMR experiments were performed. All the experiments were conducted using DMSO-d_6_ as a solvent at 25 °C. The pulse sequences were obtained from the library of the spectrometer and the process of the spectra was analyzed through MestreNova v. 14 program [28].

### 3.3. Computational Details

Both **DKI39** and **DKI40** molecules were energetically optimized. *E* and *Z* isomers, as well as *endo* and *exo* conformers, were computed employing the Density Functional Theory [29] at the B3LYP [30,31]/6-311 + G(d,p) [32] level of theory. All calculations were performed in DMSO solvent employing the polarizable continuum model (PCM) [33,34]. It has been shown that the present DFT methodology is adequate for the prediction of different isomers and conformers [35,36,37,38]. The transition states connecting the various minima structures were calculated using the synchronous transit-guided quasi-Newton (STQN) method [39]. Vibrational analysis and IRC calculations confirmed that they are transition states. The calculations and the visualization of the results were performed through Gaussian 16 [40].

### 3.4. Molecular Binding

Docking [41] experiments were performed using Autodock [42] software and Lamarckian genetic algorithm. The crystal structures of the macromolecules were downloaded from the Protein Data Bank (PDB) platform and the compounds were minimized with MM2 force field.

### 3.5. Molecular Dynamics

Explicit solvent atomistic simulation systems were prepared from DFT-optimized structures of ligand–receptor complexes using the online server CHARMM-GUI [43]. Both systems were electroneutralized in 150 mM NaCl, and the TIP3P [44] water model was used. We chose a cubic simulation box with the side length of 8.5 nm. Systems were energy-minimized using the steepest descent and the adaptive basis Newton–Raphson method. Both systems were exposed to an equilibration phase of 10 ns. All simulations were carried out on GPUs with the CUDA version of the NAMD [45] (ver. 2.13) MD software suite. The CHARMM36 [46] force field was used, which included parameters for protein, water and ions. Missing parameters for the ligands were derived using the Paramchem [47] server, while the partial atomic charges were further refined by ab initio calculation performed by the Gaussian suit of programs [48]. All production simulations used the constant number of particles, pressure and temperature (NPT) ensemble and each run was 300 ns long. Temperature was held constant (303.15 K) using the Langevin thermostat with a dampening constant of 1 ps^−1^, and the pressure was held constant at 1 bar using the Nose–Hoover Langevin piston for pressure control. The time-step of the production simulations was 2 fs and the SHAKE algorithm was used for hydrogen atoms. The cutoff for non-bonded interactions was set to 12 Å, and electrostatic interactions were calculated using the Particle Mesh Ewald method [49].

### 3.6. Pharmacokinetic and Toxicity Properties

Both compounds were examined for their drug-likeness and toxicity through SwissADME [15], pkCSM [50] and preADMET [51]. This is very important because many biological compounds fail due to unfavorable ADME results.

## 4. Conclusions

**Scheme 9:**
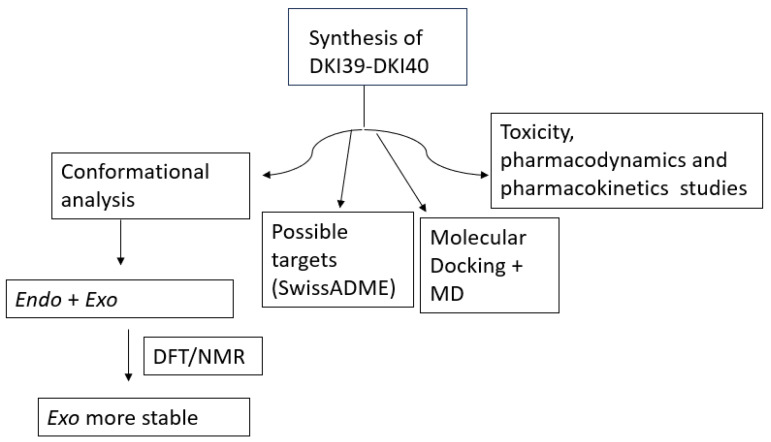
Summary of the scientific work described in the manuscript.

Scheme 9 includes all the research activities applied in the manuscript.

In particular, the present study focused on the synthesis of two thiazolidine-4-ones and on their study via DFT methodology and NMR spectroscopy. The aim of this study was to decipher which isomers and conformers are the most stable ones, as well as to check if the isomers or conformers can easily convert one to another.

DFT showed that these two molecules can form stable *endo* and *exo* conformations, but the *exo* ones are the most stable in energy, in agreement with our 2D-NMR measurements. The calculations of the reaction isomerization paths, and the calculation of the corresponding transition states, confirm our NMR results that for the *exo* compounds, only one conformation is synthesized.

In silico experiments of molecular docking were performed on five macromolecules, i.e., triazoloquinazolines, mglur3, Jak3, Danio rerio HDAC6 CD2 and acetylcholinesterase. The results showed that the compounds bind strongly to them and both compounds obey Lipinski’s Rule of Five and they are not hepatotoxic. The molecular dynamics data showed that both compounds are stable in acetylcholinesterase and in metabotropic glutamate receptor 3 for DKI40 and DKI39, respectively.

To sum up, the present study showed that thiazolidine-4-ones are stable, safe and bioactive in various targets. This study aimed to investigate the physical and chemical properties of two synthetic analogues and examine the possibility of them serving as lead compounds for various diseases. These conclusions are quite important for synthetic organic scientists who synthesize similar derivatives and test them as potential biological targets.

## Figures and Tables

**Table 1:** Assignment of the experimental ^1^H NMR spectra of **DKI39** and **DKI40** in DMSO-d_6_.

**Hydrogen DKI39**	**Chemical Shift (ppm)**	**Hydrogen DKI39**	**Chemical Shift (ppm)**
1	7.68	12	7.63
2	7.50	14	7.47
3	7.85	15	7.57
5	8.53	16	7.47
**Hydrogen DKI40**	**Chemical Shift (ppm)**	**Hydrogen DKI40**	**Chemical Shift (ppm)**
1	7.36	12	7.58
2	7.50	14	7.62
3	7.83	15	7.12
5	8.40	18	3.86

**Table 2:** Relative energies (ΔΕ in kcal/mol) and dihedral angles (τ in degrees) of the minimized structures for **DKI39** *endo* and **DKI40** *endo* compounds ^a^.

**DKI39 *endo***	**ΔΕ**	**τ_2_**	**τ_1_**	**τ_3_**
DKI39en_1	0	*Z* (180.0)	*E* (−180.0)	-
DKI39en_2	4.79	*Z* (180.0)	*Z* (−2.2)	-
DKI39en_3	7.91	*E* (0.8)	*E* (−179.9)	-
DKI39en_4	9.71	*E* (−0.3)	*Z* (2.2)	-
**DKI40 *endo***	**ΔΕ**	**τ_3′_**	**τ_1′_**	**τ_2′_**
DKI40en_1	0	*Z* (180.0)	*E* (180.0)	-
DKI40en_3	4.63	*E* (0.1)	*E* (180.0)	-
DKI40en_4	5.00	*Z* (−180.0)	*Z* (2.2)	-
DKI40en_2	9.39	*E* (0.1)	*Z* (−2.03)	-

^a^ ΔΕ Difference in energy between the various conformers and the minimum conformer; τ_1_, Amide Bond Isomerization (5-6) C-C=N-C with dihedral angle; τ_2_, Double Bond Isomerization C-C=C-C with dihedral angle; τ_3_, Amide Bond Isomerization (7-8) N-N=C-N with dihedral angle; τ_1′_, Amide Bond Isomerization (5-6) C-C=N-C with dihedral angle; τ_2′_, Amide Bond Isomerization (7-8) N-N=C-C with dihedral angle; τ_3′_, Double Bond Isomerization (11-12) C-C=C-C with dihedral angle.

**Table 3:** Relative energy differences of the conformers with the respect to the global minimum, ΔΕ, and calculated dihedral angles (τ_1_, τ_2_, τ_3_) for **DKI39** *exo* and **DKI40** *exo* compounds ^a^.

**DKI39 *exo***	**ΔΕ**	**τ_2_**	**τ_1_**	**τ_3_**
DKI39ex_1	0	*Z* (180.0)	*Z*(−180.00)	*E* (−180.00)
DKI39ex_4	1.54	*Z* (−180.0)	*E* (180.0)	*E* (−0.0)
DKI39ex_2	3.98	*Z* (180.0)	*Z* (−0.2)	*Z* (180.0)
DKI39ex_8	5.17	*E* (0.0)	*Z* (−180.0)	*E* (−180.0)
DKI39ex_3	5.59	*Z* (180.0)	*E* (−1.5)	*Z* (−1.53)
DKI39ex_5	6.67	*E* (0.16)	*E* (−180.0)	*E* (0.0)
DKI39ex_7	9.09	*E* (0.0)	*Z* (−0.11)	*Z* (180.0)
DKI39ex_6	10.71	*E* (0.1)	*E* (−0.0)	*Z* (−0.0)
**DKI40 *exo***	**ΔΕ**	**τ_3′_**	**τ_1′_**	**τ_2′_**
DKI40ex_1	0	*Z* (180.0)	*Z* (180.0)	*E* (0.0)
DKI40ex_4	1.53	*Z* (−0.0)	*E* (180.0)	*E* (−0.0)
DKI40ex_2	3.95	*Z* (−180.0)	*Z* (−0.0)	*Z* (180.0)
DKI40ex_6	4.75	*E* (−0.0)	*Z* (−180.0)	*E* (180.0)
DKI40ex_3	5.69	*Z* (180.0)	*E* (−0.9)	*Z* (−0.9)
DKI40ex_5	6.40	*E* (0.0)	*E* (180.0)	*E* (−0.0)
DKI40ex_8	8.63	*E* (0.0)	*Z* (0.0)	*Z* (180.0)
DKI40ex_7	10.52	*E* (−180.0)	*E* (−0.04)	*Z* (180.0)

^a^ Difference in energy between the various conformers and the minimum conformer; ΔΕ difference in energy between the various conformers and the minimum conformer; τ_1_, Amide Bond Isomerization (5-6) C-C=N-C with dihedral angle; τ_2_, Double Bond Isomerization C-C=C-C with dihedral angle; τ_3_, Amide Bond Isomerization (7-8) N-N=C-C with dihedral angle; τ_1′_, Amide Bond Isomerization (5-6) C-C=N-C with dihedral angle; τ_2′_, Amide Bond Isomerization (7-8) N-N=C-C with dihedral angle; τ_3′_, Double Bond Isomerization (11-12) C-C=C-C with dihedral angle.

**Table 4:** Selected H-H distances in Å at DFT.

DKI39b	H-H	DKI40a	H-H
H12-H14	2.280	H18-H15	2.352
H5-H3	2.354	H5-H3	2.373

**Table 5:** Molecular docking results for each compound with the macromolecules.

Binding Energy (kcal/mol)		
Macromolecules	DKI39	DKI40
2Y0J (Triazoloquinazolines)	−8.22	−7.82
5CNK (mglur3)	−9.16	−6.19
5TTV (Jak3)	−8.95	−8.83
8A8Z (Danio rerio HDAC6 CD2)	−8.23	−7.94
4EY7 (Acetylcholinesterase)	−8.95	−8.92

**Table 6:** Drug-likeness of the compounds.

Properties	Compound DK139	Compound DKI40
Molecular Weight	307.37	337.40
LogP	2.66	3.08
Rotable bonds	3	4
Hydrogen Bond Acceptors	3	4
Hydrogen Bond Donors	1	1
Surface Area	79.12 (Å^2^)	88.35 (Å^2^)
Water solubility	−4.54 (mol/L)	−4.61 (mol/L)

**Table 7:** ADME results according to preADMET.

	Compound DKI39	Compound DKI40
BBB	0.565933	0.304397
Buffer_solubility_mg_L	171.672	163.776
Caco2	22.0759	21.9635
CYP_2C19_inhibition	Non	Non
CYP_2C9_inhibition	Non	Non
CYP_2D6_inhibition	Non	Non
CYP_2D6_substrate	Non	Non
CYP_3A4_inhibition	Non	Non
CYP_3A4_substrate	Weakly	Weakly
HIA	96.387179	96.785453
MDCK	11.204	2.28162
Pgp_inhibition	Inhibitor	Inhibitor
Plasma_Protein_Binding	94.011663	93.567531
Pure_water_solubility_mg_L	0.562912	0.382868
Skin_Permeability	−2.82467	−3.03751

**Table 8:** Toxicity results for both compounds according to pKCSm.

Properties	Compound DKI39	Compound DKI40
Toxicity		
AMES toxicity	Yes	Yes
Max. tolerated dose (human)	0.064 (log mg/kg/day)	0.074 (log mg/kg/day)
Herg I inhibitor	No	No
Herg II inhibitor	Yes	No
Oral rat acute toxicity (LD50)	2.318 (mol/kg)	2.268 (mol/kg)
Oral rat chronic toxicity	1.432 (log mg/kg_bw/day)	1.723 (log mg/kg_bw/day)
Hepatotoxicity	No	No
Skin sensitization	No	No

## Data Availability

The data presented in this paper are available in the Supplementary Materials.

## Supplementary Materials

The following supporting information can be downloaded at: https://www.mdpi.com/article/10.3390/molecules29112458/s1, Figure S1A: ^1^H spectra of DKI39. The spectra were recorded in DMSO-d_6_ on a Bruker AC 800 MHz spectrometer at 25 °C; Figure S2A: 2D-NOESY spectra of DKI39. The spectra were recorded in DMSO-d_6_ on a Bruker AC 800 MHz spectrometer at 25 °C; Figure S3A: ^13^C spectra of DKI39. The spectra were recorded in DMSO-d_6_ on a Bruker AC 800 MHz spectrometer at 25 °C; Figure S4A: 2D-HSQC spectra of DKI39. The spectra were recorded in DMSO-d6 on a Bruker AC 800 MHz spectrometer at 25 °C; Figure S5A: 2D-HSQC spectra of DKI39. The spectra were recorded in DMSO-d6 on a Bruker AC 800 MHz spectrometer at 25 °C; Figure S1B: 1H spectra of DKI40. The spectra were recorded in DMSO-d6 on a Bruker AC 800 MHz spectrometer at 25 °C; Figure S2B: 13C spectra of DKI40. The spectra were recorded in DMSO-d6 on a Bruker AC 800 MHz spectrometer at 25 °C; Figure S3B: 2D-NOESY spectra of DKI40. The spectra were recorded in DMSO-d6 on a Bruker AC 800 MHz spectrometer at 25 °C; Figure S4B: 2D-HSQC spectra of DKI40. Figure S5B: 2D-HMBC spectra of DKI40. The spectra were recorded in DMSO-d6 on a Bruker AC 800 MHz spectrometer at 25 °C; Table 1S: Energetic isomerization between *cis-trans* for DKI39exo; Table 2S: Energetic isomerization between *cis-trans* for DKI40exo.
