# Supplementary material for: Synthesis of Thiazolidin-4-Ones Derivatives, Evaluation of Conformation in Solution, Theoretical Isomerization Reaction Paths and Discovery of Potential Biological Targets
Source: Molecules. 2024 May 23;29(11):2458. doi: 10.3390/molecules29112458 (PMC11173912; doi:10.3390/molecules29112458)
Supplement: Supplementary file 1 [file molecules-29-02458-s001.zip › molecules-3002137-supplementary.pdf]

Synthesis of thiazolidin-4-ones derivatives, 2D-NMR spectra, theoretical isomerization reaction paths and discovery of potential biological targets

Nikitas Georgiou [1], Danai Karta [1], Antigoni Cheilari [2], Franci Merzel [3], Demeter Tzeli [4,5,*], Stamatia Vassiliou [1,*], Thomas Mavromoustakos [1,*]

[1]*National and Kapodistrian University of Athens, Department of Chemistry, Laboratory of Organic Chemistry, Panepistimioupolis Zografou, 11571, Athens*
[2]*Department of Pharmacognosy and Natural Products Chemistry, Faculty of Pharmacy, National and Kapodistrian University of Athens, Panepistimiopolis Zografou, 15771 Athens, Greece*
[3]*Theory Department, National Institute of Chemistry, 1000 Ljubljana, Slovenia.*
[4]*National and Kapodistrian University of Athens, Department of Chemistry, Laboratory of Physical Chemistry, Panepistimioupolis Zografou, 11571, Athens*
[5]*Theoretical and Physical Chemistry Institute, National Hellenic Research Foundation, 48 Vassileos Constantinou Ave., Athens 116 35, Greece*

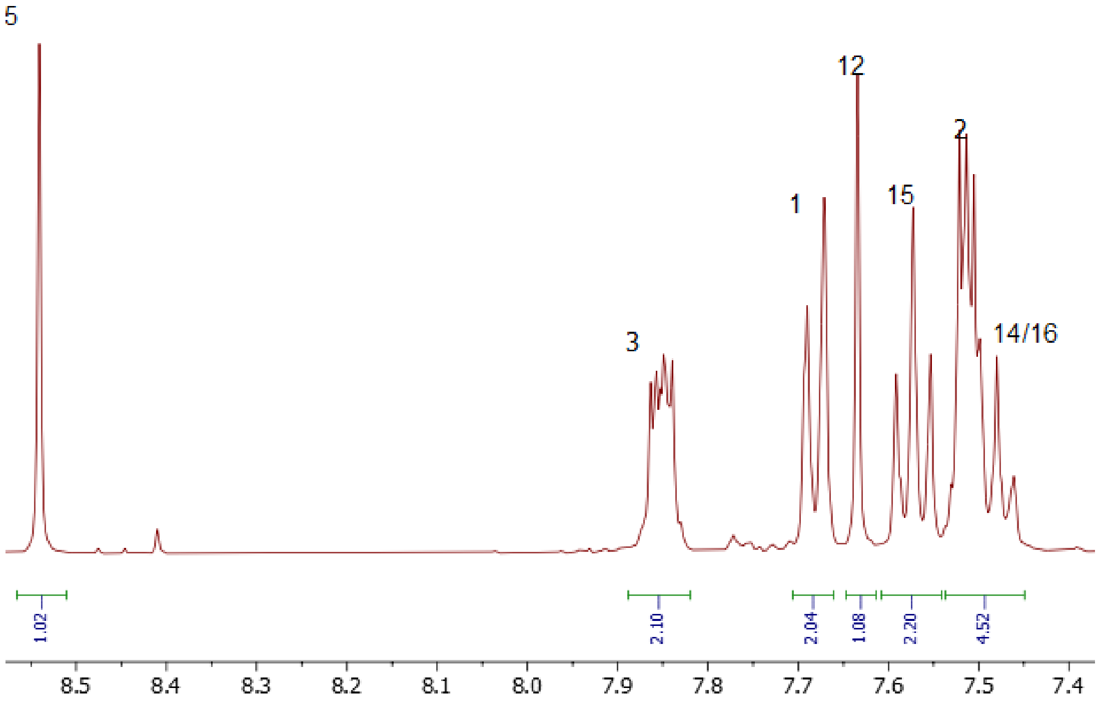

Figure S1A. ^{1}H spectra of DKI39. The spectra were recorded in DMSO-d_6 on a Bruker AC 400MHz spectrometer at 25°C.

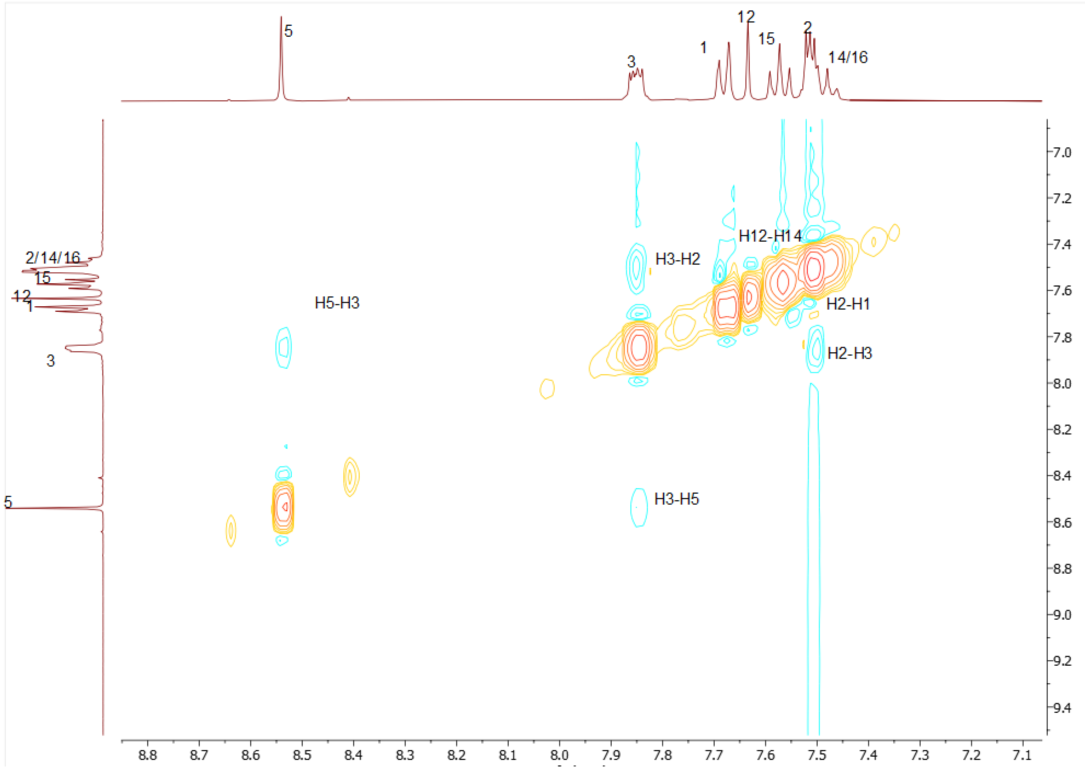

Figure S2A. 2D-NOESY spectra of DKI39. The spectra were recorded in DMSO-d_6 on a Bruker AC 400MHz spectrometer at 25°C.

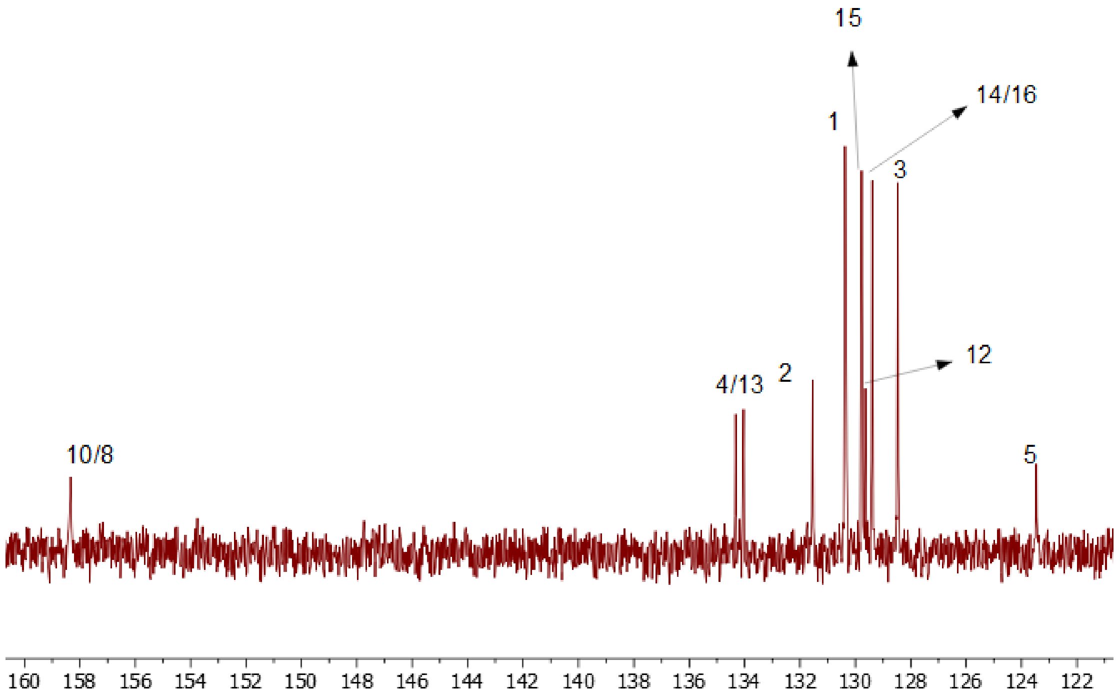

Figure S3A. ^{13}C spectra of DKI39. The spectra were recorded in DMSO-d_6 on a Bruker AC 400MHz spectrometer at 25°C.

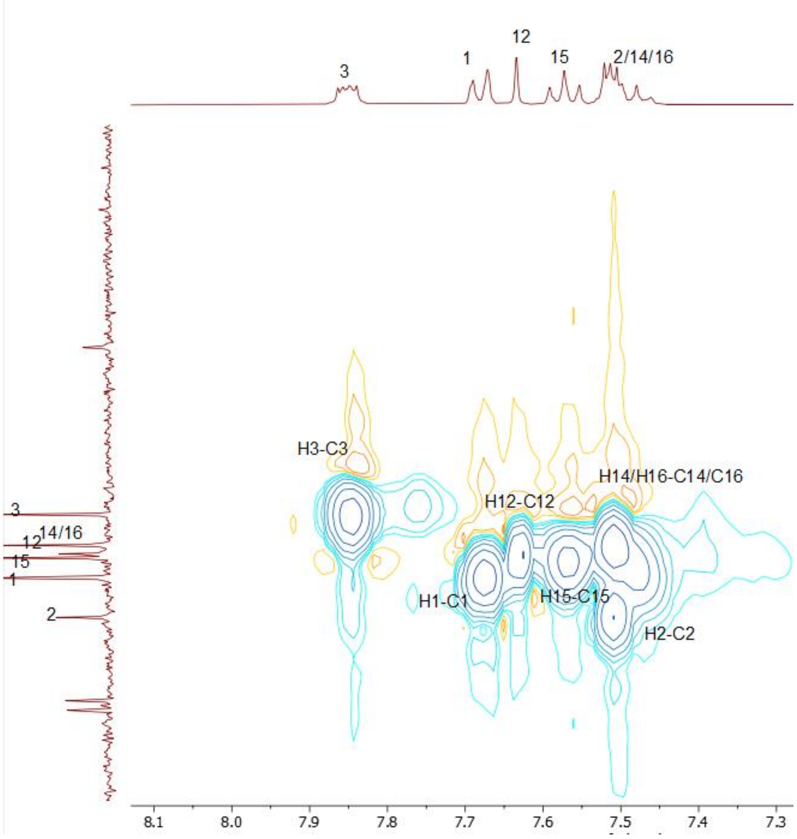

Figure S4A. 2D-HSQC spectra of DKI39. The spectra were recorded in DMSO-d_6 on a Bruker AC 400MHz spectrometer at 25°C.

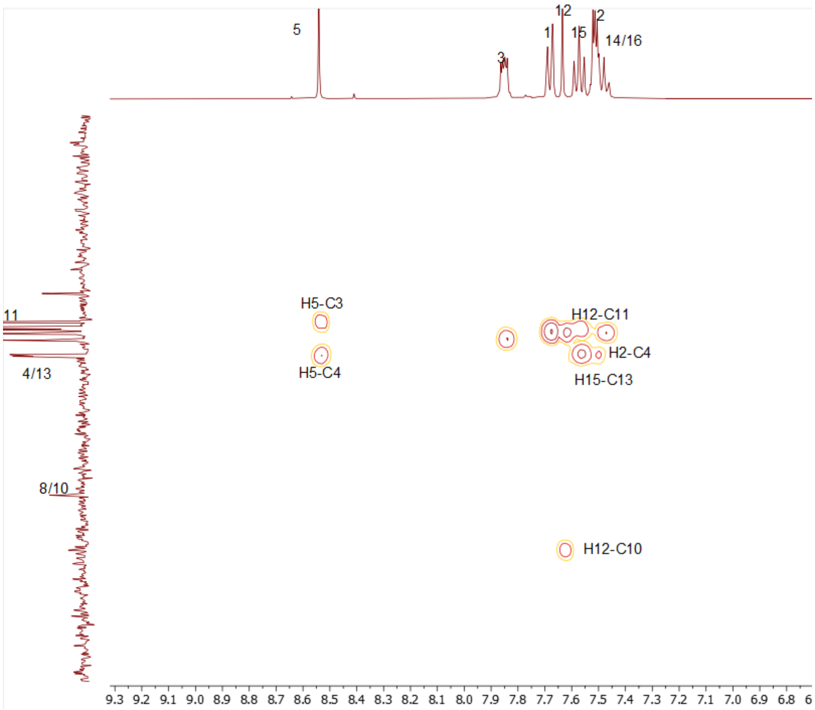

Figure S5A. 2D-HSQC spectra of DKI39. The spectra were recorded in DMSO-d_6 on a Bruker AC 400MHz spectrometer at 25°C.

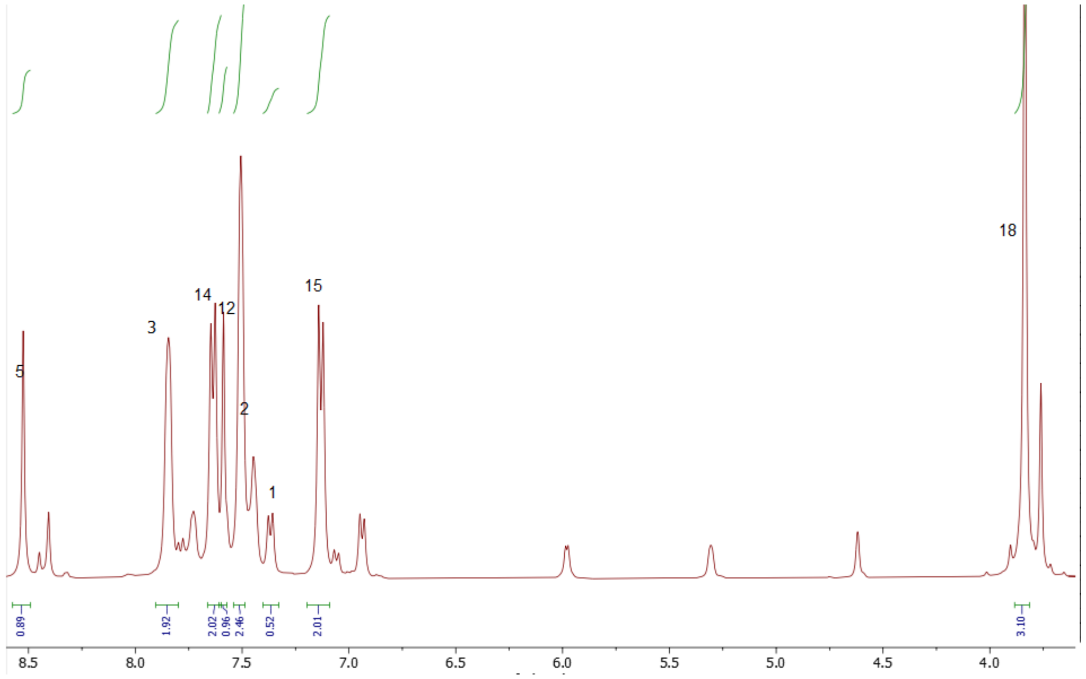

Figure S1B. ^{1}H spectra of DKI40. The spectra were recorded in DMSO-d_6 on a Bruker AC 400MHz spectrometer at 25°C.

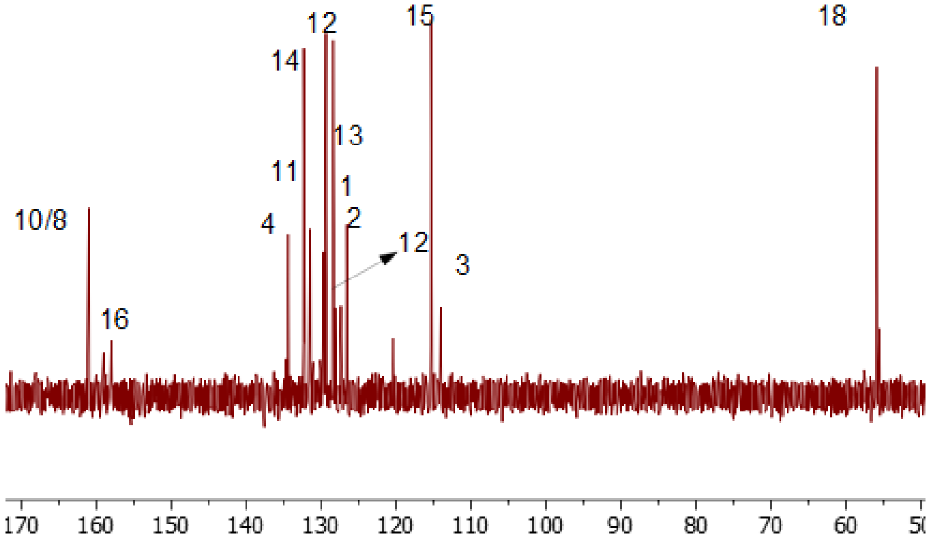

Figure S2B. ^{13}C spectra of DKI40. The spectra were recorded in DMSO-d_6 on a Bruker AC 400MHz spectrometer at 25°C.

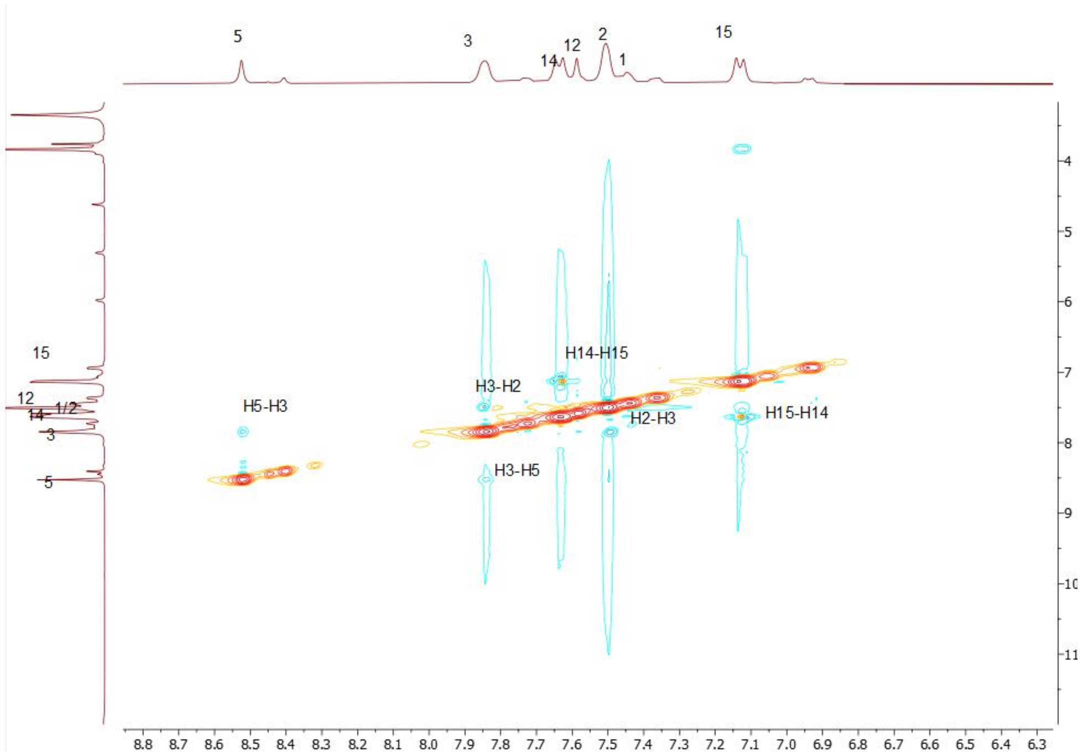

Figure S3B. 2D-NOESY spectra of DKI40. The spectra were recorded in DMSO-d_6 on a Bruker AC 400MHz spectrometer at 25°C.

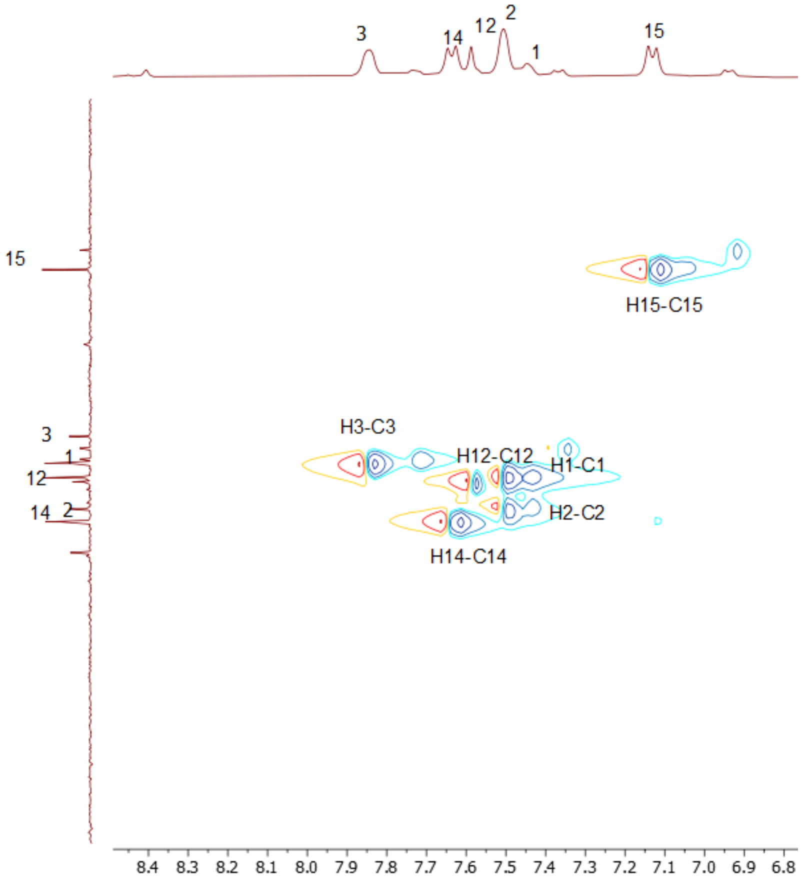

Figure S4B. 2D-HSQC spectra of DKI40. The spectra were recorded in DMSO-d_6 on a Bruker AC 400MHz spectrometer at 25°C.

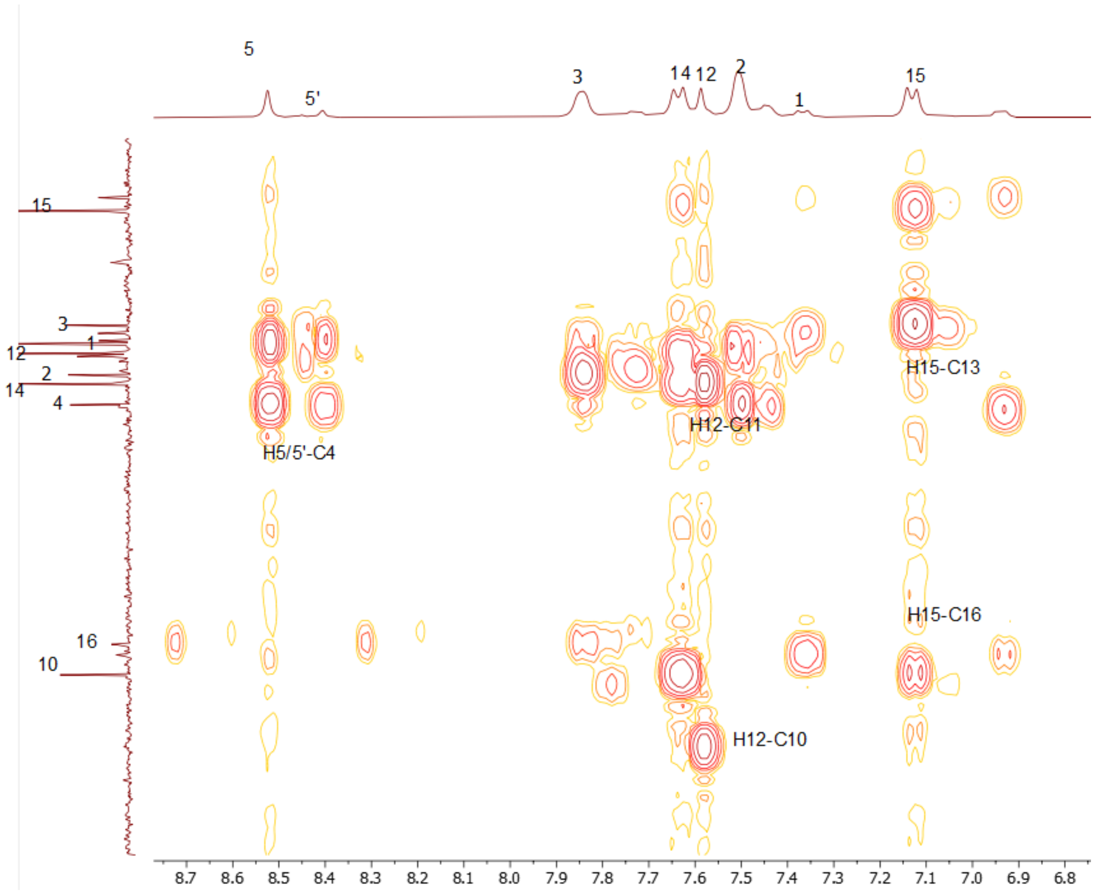

Figure S5B. 2D-HMBC spectra of DKI40. The spectra were recorded in DMSO-d_6 on a Bruker AC 400MHz spectrometer at 25°C.

Table S1: Energetics isomerization between *cis-trans* for DKI39exo

Isomer	Energy (Hartree)	Relative Energy (kcal/mol)
DKI39ex_1	-1293.56079	0.00
TS1	-1293.505585	34.64
DKI39ex_2	-1293.554448	3.98
TS2	-1293.518151	26.76
DKI39ex_3	-1293.551881	5.59
TS3	-1293.504212	35.50
DKI39ex_5	-1293.558337	1.54
TS4	-1293.53985	13.14
DKI39ex_8	-1293.550154	6.67
TS5	-1293.494687	41.48
DKI39ex_4	-1293.543723	10.71
TS6	-1293.509831	31.98
DKI39ex_7	-1293.546312	9.09
TS7	-1293.496534	40.32
DKI39ex_6	-1293.552552	5.17

Table S2: Energetic isomerization between *cis-trans* for DKI40exo

Isomer	Energy (Hartree)	Relative Energy (kcal/mol)
DKI40ex_1	-1408.121245	0.00
TS1	-1408.065344	35.08
DKI40ex_2	-1408.114952	3.95
TS2	-1408.078518	26.81
DKI40ex_8	-1408.112174	5.69
TS3	-1408.063951	35.95
DKI40ex_7	-1408.118806	1.53
TS4	-1408.071347	31.31
DKI40ex_6	-1408.111045	6.40
TS5	-1408.065344	35.08
DKI40ex_5	-1408.104488	10.52
TS6	-1408.070763	31.68
DKI40ex_4	-1408.107491	8.63
TS7	-1408.056662	40.53
DKI40ex_3	-1408.113672	4.75
